# Proximity Proteomics Maps Candidate Cellular Uptake Pathways for Cell‐Derived Nanovesicles

**DOI:** 10.1002/smtd.71014

**Published:** 2026-09-02

**Authors:** Eisuke Kanao, Ryosuke Mizuta, Saki Tarao, Yukika Hara, Yuka Li, Kazunari Akiyoshi, Yasushi Ishihama, Koshi Imami, Yoshihiro Sasaki

**Affiliations:** ^1^ Graduate School of Pharmaceutical Sciences Kyoto University Kyoto Japan; ^2^ Laboratory of Proteomics for Drug Discovery National Institute of Biomedical Innovation Health and Nutrition Ibaraki Osaka Japan; ^3^ Graduate School of Engineering Kyoto University Kyoto Japan; ^4^ Graduate School of Medicine Kyoto University Kyoto Japan; ^5^ Proteome Homeostasis Research Unit RIKEN Center for Integrative Medical Sciences Yokohama Kanagawa Japan

**Keywords:** cell‐derived nanovesicle, cellular uptake pathway, extracellular vesicle, proximity labelling

## Abstract

Although extracellular vesicles (EVs) facilitate selective molecular exchange between cells, their low yields and inherent heterogeneity limit translational applications. Cell‐derived nanovesicles (CDNs), produced by mechanical extrusion of donor cells, offer a scalable alternative while retaining key membrane features of EVs. Yet how fabrication reshapes vesicle–cell communication remains poorly understood. Here, vesicle‐side proteomics is integrated with TurboID proximity labeling of recipient‐cell proteins to construct quantitative, dual‐sided maps of CDN and small extracellular vesicle (sEV) interactions. Despite similar size and surface charge, CDNs displayed a substantially more diverse proteome and a broader repertoire of predicted uptake‐associated signatures. Recipient‐cell proximity proteomics further resolved distinct molecular cohorts. The CDN‐associated cohort contained recipient‐derived CALR and NCL, which are literature‐linked to LRP1/CD91‐associated efferocytic recognition and NCL‐associated macropinocytic processes, respectively, whereas the sEV‐associated cohort contained signatures consistent with HSPG‐assisted docking and clathrin‐mediated or CLIC/GEEC‐related uptake. Together, these complementary datasets reveal molecular interfaces for extrusion‐generated CDNs that are distinct from those of naturally secreted sEVs. Receptor dependence and the contributions of surface association and internalization require direct testing; however, the identified associations define specific mechanistic targets for further investigation. This dual‐sided proteomic strategy establishes a quantitative framework for dissecting vesicle–cell communication and engineering membrane‐based nanocarriers.

## Graphical abstract

TurboID‐based dual‐sided proteomics compares mechanically generated cell‐derived nanovesicles with naturally secreted small extracellular vesicles. Vesicle proteomes define candidate interaction signatures, while recipient‐cell proximity labelling identifies exposure‐associated proteins. Together, the complementary datasets reveal shared and condition‐associated molecular features across both systems, prioritizing testable vesicle–cell interactions for mechanistic validation and nanocarrier engineering.

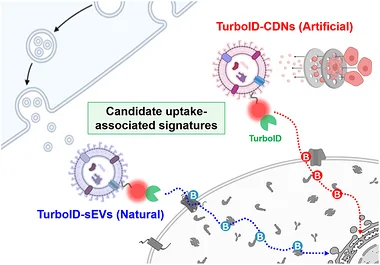

## 1 Introduction

Extracellular vesicles (EVs) are lipid‑bilayer nanoparticles, typically measuring 30–200 nm in diameter, that selectively transport molecular cargo—including proteins, lipids, and nucleic acids—from donor to recipient cells. Consequently, EVs are responsible for mediating intercellular communication and have the ability to reprogram distant microenvironments [1, 2, 3]. Despite the weakly immunogenic nature of their endogenous membranes, EVs retain surface ligands that enable tissue‑specific homing, thereby positioning them as attractive carriers for regenerative medicine and drug delivery [4, 5, 6].

However, the clinical translation of EVs is limited by three intrinsic constraints. Firstly, their yields from producer cells are generally low; for instance, human mesenchymal stromal cells (MSCs) typically secrete only 1–4 µg of EV‐associated proteins per 10^6^ cells in a 24 h period under standard two‐dimensional culture conditions [7]. At the particle level, this corresponds to roughly 1–4 × 10^8^ EVs per 10^6^ cells, equivalent to ∼100–400 EVs per cell over 24 h, consistent with nanoparticle‐tracking measurements of MSC‐derived EVs [8]. Given that preclinical mouse studies typically require 10–30 µg per dose and early‐phase clinical trials demand hundreds of micrograms, manufacturing costs are extremely high [9, 10]. Second, isolation is labor‐intensive: density‐gradient or ultracentrifugation protocols entail multi‐hour, multi‐step workflows yet recover less than 25% of total vesicles [11, 12]. Thirdly, natural EVs are heterogeneous [13]; vesicles arising from distinct biogenic routes differ with respect to their cargo and function [14], and donor‑to‑donor variability further amplifies differences between batches, despite controlled culture conditions [15]. Collectively, these issues hinder scalability and standardization.

To address these limitations, cell‑derived nanovesicles (CDNs) have emerged as a pragmatic alternative to EVs [16, 17]. CDNs are produced by mechanically disrupting cultured cells, which allows plasma membranes to undergo subsequent self‐assembly into vesicles. Systematic comparisons have shown that CDN yields tend to be 10–100 times higher than those of matched EV preparations, significantly reducing production times and lowering costs [18, 19]. Current fabrication methods for CDNs include nitrogen cavitation [20], probe ultrasonication [21], high‐pressure homogenization [22], and microfluidic shear processing [23]. Among these, sequential extrusion through polycarbonate membranes is particularly attractive as it enables particle size homogenization and scalability to larger volumes [24, 25, 26]. Notably, CDNs preserve the native membrane architecture and cytosolic components of the parent cells and therefore are expected to exhibit EV‐like delivery competence and homing behavior. Related studies of apoptotic bodies have shown that parent‐cell manipulation, cargo loading, and post‐isolation surface modification can tune the therapeutic behavior of cell‐derived vesicles while also altering biological recognition and cellular uptake [27].

Despite such promise, the impact of fabrication on the molecular mechanisms of vesicle–cell interactions remains poorly understood. Although EVs and CDNs often share bulk physicochemical properties (e.g., diameter and zeta potential), mechanical inputs during their fabrication can alter the composition, abundance, and orientation of membrane proteins, potentially shifting the balance among uptake routes, including the clathrin‑mediated, clathrin‑independent, adhesion‑driven, and macropinocytic pathways [28, 29, 30, 31, 32, 33]. At present, conventional fluorescence imaging and inhibitor screening provide only qualitative snapshots and are confounded by pathway crosstalk and off‑target effects [34, 35, 36]. Accordingly, a proteome‐level map could relate artificial vesicle composition to candidate uptake‐associated signatures.

In this context, the combination of proximity‑dependent biotinylation with mass spectrometry (MS) provides a means to chart protein–protein interactions in living cells. For example, Ren et al. tracked the intracellular trafficking of horseradish peroxidase (HRP)‑functionalized poly(lactic‐co‐glycolic acid) nanoparticles using proximity proteomics, mapping their sequential endosomal–lysosomal–endoplasmic reticulum–Golgi itineraries [37]. Among the available labeling enzymes, TurboID, an engineered Escherichia coli biotin ligase, produces reactive biotin‑5′‑AMP and labels lysine residues within a distance of ∼10 nm, exhibiting >100‑fold higher catalytic efficiency than earlier proximity‐labeling methods [38, 39]. Previously, our group introduced TurboID–mCherry into sEVs derived from HEK293T cells and catalogued 456 recipient‑cell proteins that transiently associate with sEVs [40].

In the current study, this platform is extended to extrusion‐generated CDNs, providing a systems‐level comparison of how proteome differences between similar‑sized CDNs and sEVs relate to candidate uptake‐associated signatures (Figure 1). The framework is intended to generate testable mechanistic hypotheses, thereby supporting the rational design of cell‐derived and membrane‐hybrid nanocarriers.

**FIGURE 1:**
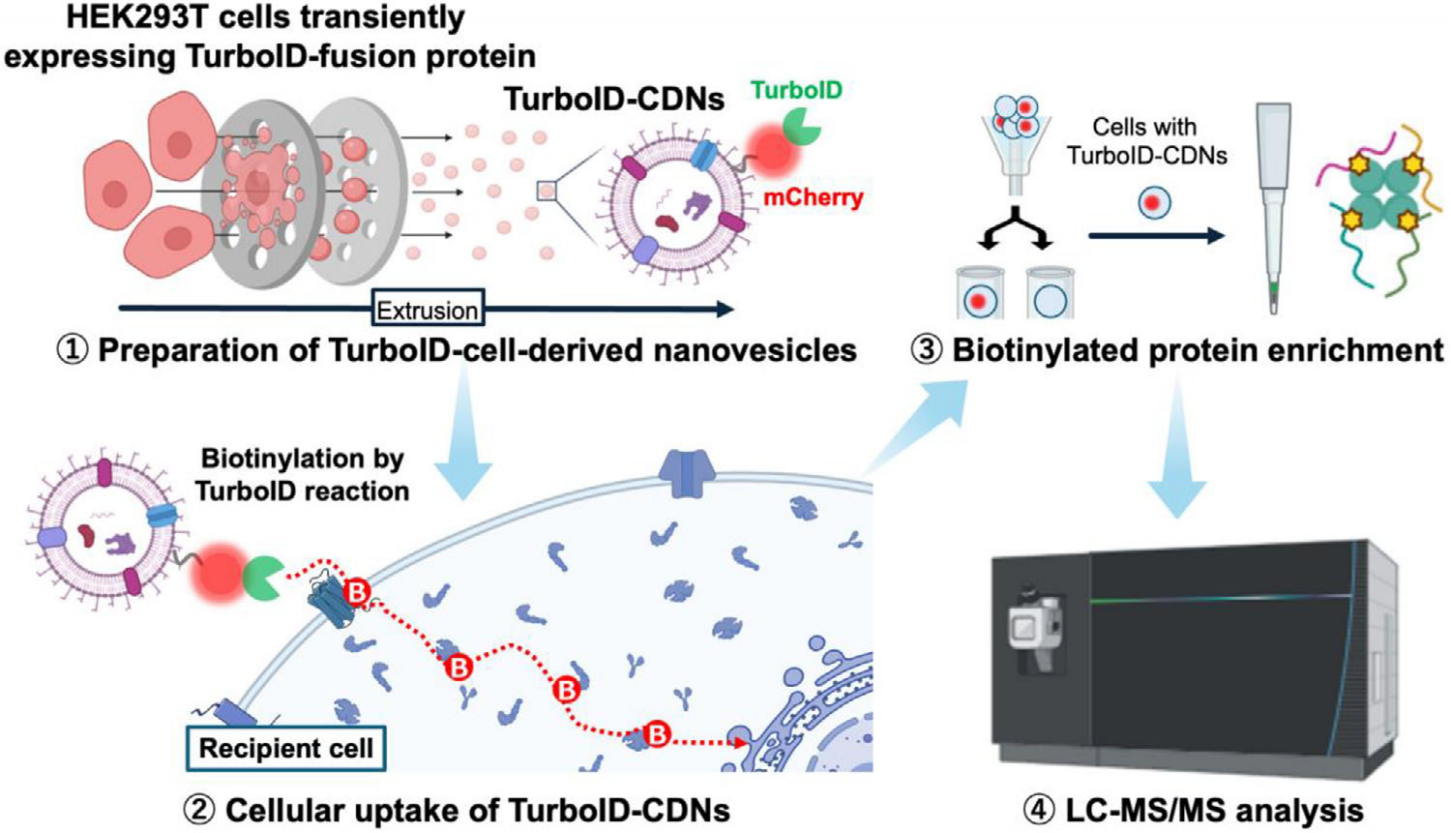
TurboID‐based proximity proteomics workflow for CDNs. HEK293T cells transiently expressing MFGE8(D49E)–mCherry–TurboID are mechanically disrupted and sequentially extruded to produce TurboID‐CDNs. After incubation with recipient cells in the presence of biotin, vesicle‐associated TurboID biotinylates proteins proximal to the vesicle–cell interface, potentially during cell‐surface association and/or after internalization. mCherry‐positive recipient cells are isolated by FACS and lysed. The biotinylated proteins are enriched on streptavidin resin, digested, and analyzed by LC‐MS/MS to generate candidate recipient‐cell proximity profiles associated with exposure to TurboID‐CDN preparations. Some graphical elements in this figure were created with BioRender.com.

## 2 Results and Discussion

### 2.1 Preparation and Physicochemical Characterization of TurboID‑CDNs

To map proteins proximal to the vesicle–cell interface, an MFGE8(D49E)–mCherry–TurboID chimera was engineered (Figure 2A), wherein the MFGE8 C1C2 domain targets phosphatidylserine‐rich membranes, while the D49E substitution reduces unintended integrin engagement by phagocytes.40 HEK293T cells transiently expressing TurboID–mCherry were harvested by scraping and were subjected to serial extrusion to produce TurboID‐CDNs. Subsequently, the crude suspension was washed with a 300 kDa cutoff ultrafiltration membrane to remove any soluble and loosely adsorbed proteins, yielding purified TurboID‐CDNs. Robust intracellular expression of TurboID–mCherry was confirmed by fluorescence microscopy (Figure 2B); purified vesicles from wild‐type cells lacked mCherry fluorescence, whereas TurboID‐CDNs showed an mCherry signal above the wild‐type CDN background, supporting incorporation of the fusion protein into the purified CDN preparation (Figure 2C). To assess the lipid‐bilayer integrity at the single‐particle level, vesicles were labeled with ExoSparkler‐Red and analyzed by imaging flow cytometry, wherein side‐scatter/bright‐field gating excluded the calibration beads and aggregates, thereby enabling quantitative fluorescence measurements. In this technical comparison, fewer fluorescent events were recorded after treatment with 0.1% Triton X‐100 than in HEPES across three technical replicates from a single TurboID‐CDN preparation (Figure 2D), consistent with detergent‐sensitive particle disruption. Additionally, nanoparticle tracking analysis (NTA) and transmission electron microscopy (TEM) confirmed the presence of uniform, spherical vesicles (Table 1; Figure S1A). The TurboID‐CDNs exhibited a mean diameter of 156.3 ± 3.8 nm and a zeta potential of −32.0 ± 0.4 mV, closely matching wild‐type HEK293T‐derived CDNs (154.3 ± 4.1 nm; −30.6 ± 1.2 mV) and similarly sized sEVs prepared by differential ultracentrifugation (131 ± 7.0 nm; −29.2 ± 3.1 mV). Importantly, these results indicate that the incorporation of TurboID does not measurably alter the vesicle size or surface charge (Table 1; Figure S1B,C).

**FIGURE 2:**
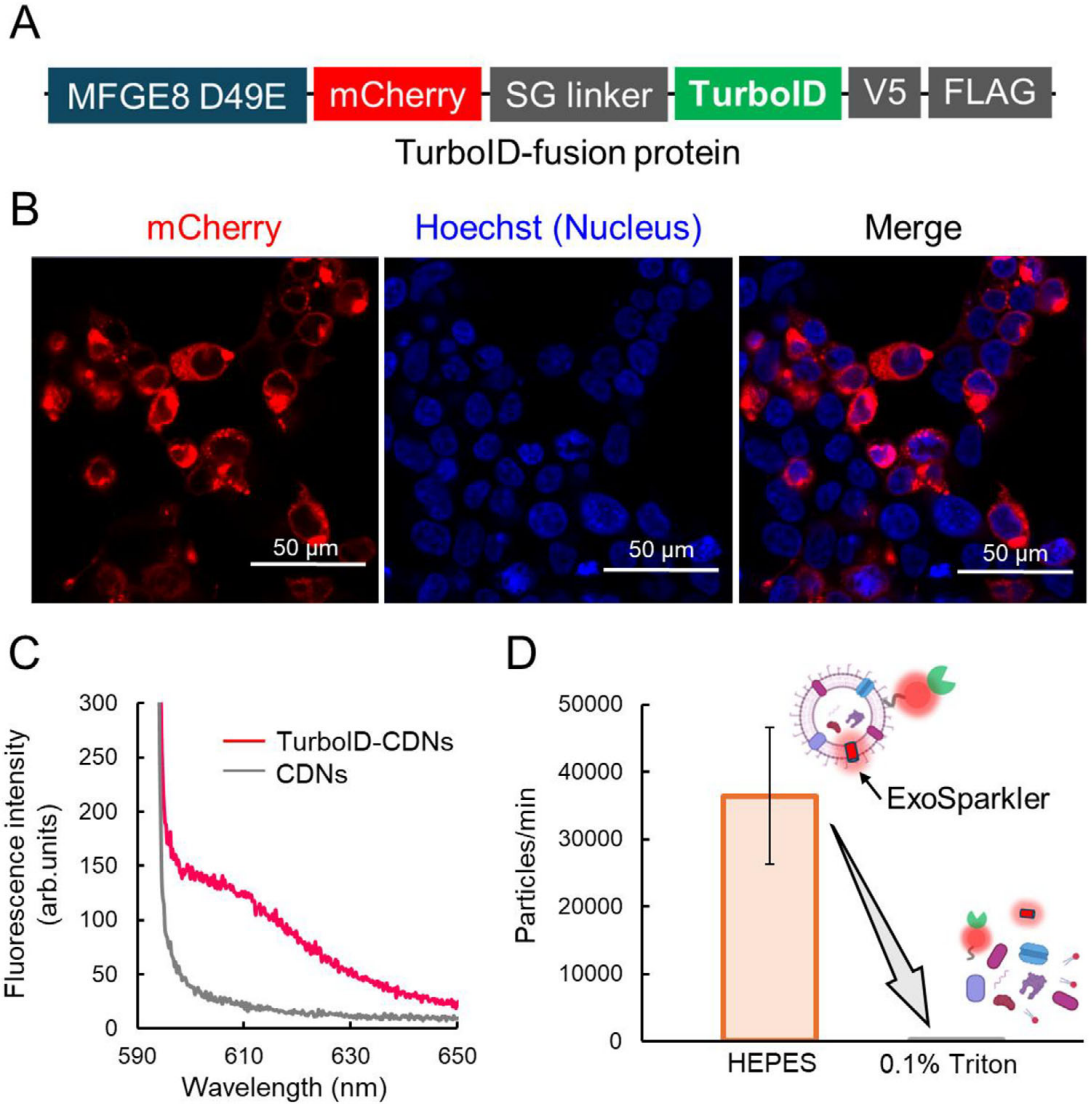
Design and validation of TurboID‑CDNs. (A) Expression cassette encoding MFGE8(D49E)–mCherry–TurboID with a C1C2 PS‐binding module, a flexible SG linker, and C‐terminal V5/FLAG tags. (B) Representative fluorescence micrographs of transiently transfected HEK293T cells (mCherry, Hoechst, merge) showing robust TurboID–mCherry expression. (C) Fluorescence intensity profiles of purified vesicles: TurboID‐CDNs show an mCherry signal above the wild‐type CDN background. (D) Imaging flow cytometry of ExoSparkler‐Red‐labeled TurboID‐CDNs. The lower particle count after treatment with 0.1% Triton X‐100 is shown as a technical detergent‐disruption control. Bars represent the mean ± SD of three paired technical replicates derived from a single TurboID‐CDN preparation. For each replicate, separate aliquots were measured in HEPES with or without 0.1% Triton X‐100.

**TABLE 1:** Sizes and surface charges of the TurboID‑CDNs, CDNs, and TurboID‑sEVs (mean ± standard deviation).

	TurboID‐CDNs	CDNs	TurboID‐sEVs
Size (nm)	156.3±3.8	154.3±4.1	131±7.0
Surface charge (mV)	−32.0±0.4	−30.6±1.2	−29.2±3.1

### 2.2 Global Proteome Profiling of TurboID‑CDNs, TurboID‑sEVs, and Their Parental Cells

TurboID‐CDNs, TurboID‐sEVs, and their parental HEK293T cells were subjected to proteome profiling using liquid chromatography–tandem MS (LC‐MS/MS; *n* = 3 biological replicates per condition). Proteins quantified in at least two of three replicates were retained for analysis. In total, 3674 proteins were identified in the cells, including 2733 in the TurboID‐CDNs and 935 in the TurboID‐sEVs (Figure 3A). Abundance‐rank analysis using intensity‐based absolute quantification placed the TurboID chimera at the extreme high‐abundance tail (top ∼3% in each sample type), supporting abundant fusion‐protein recovery in the bulk preparations (Figure S2).

**FIGURE 3:**
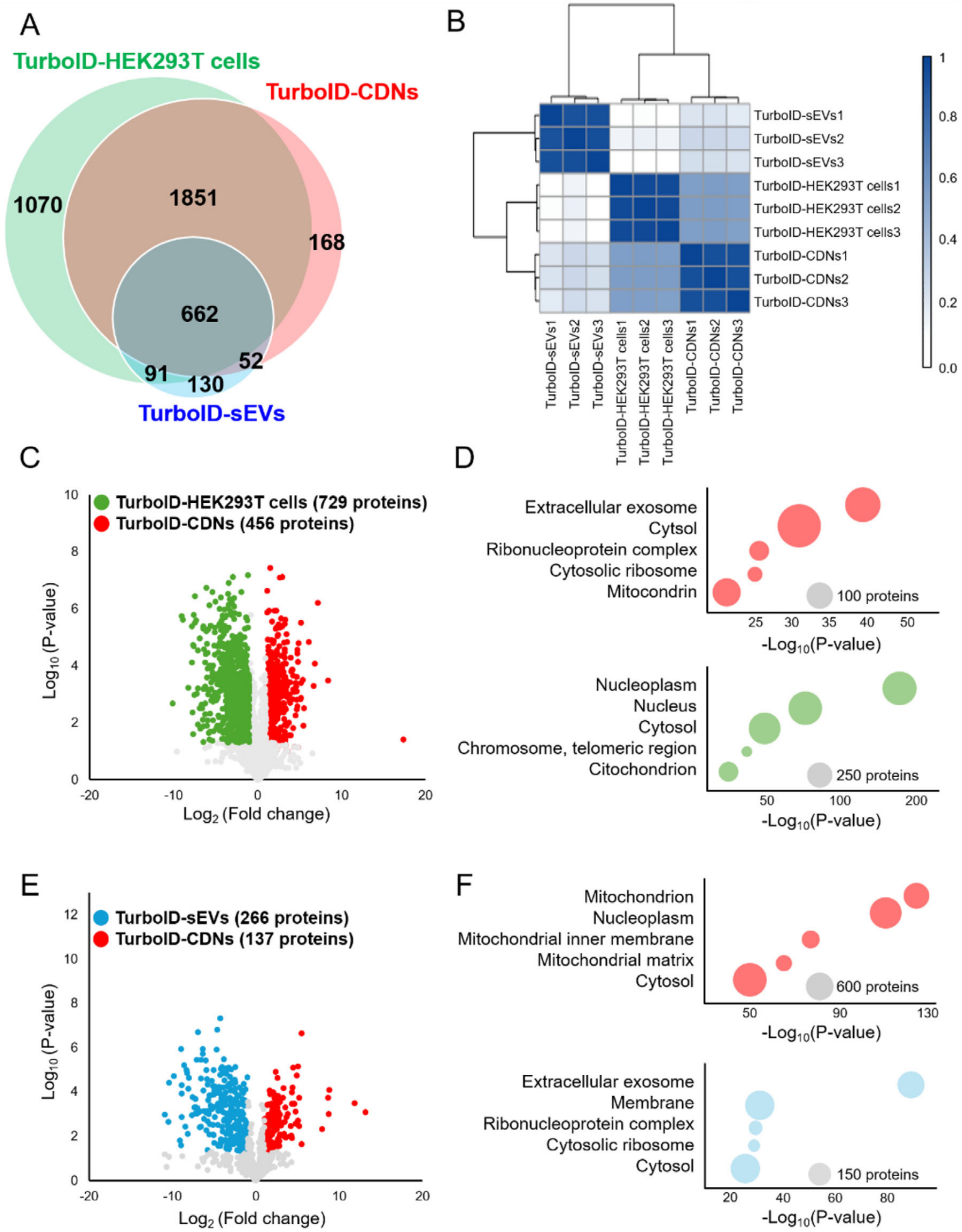
Global proteome comparison between TurboID‐CDNs, TurboID‐sEVs, and parental HEK293T cells. (A) Protein identification counts per group and overlaps among the HEK293T cells, TurboID‐CDNs, and TurboID‐sEVs. (B) Spearman correlation matrix across all biological replicates showing high within‐group reproducibility and between‐group separation. (C) Differential abundance between TurboID‐CDNs and HEK293T cells (common IDs only). Significance threshold: *fold change* ≥ 2, two‐sided *p* < 0.05. The numbers indicate the enriched proteins in each group. (D) GO Cellular Component enrichment for the CDN‐enriched combination (TurboID‐CDN unique + significantly increased). (E) Differential abundance within the CDN–sEV common set. The counts denote the enriched proteins in each vesicle type. (F) GO Cellular Component enrichment for the CDN‐enriched versus sEV‐enriched proteins, highlighting mitochondrial/nucleoplasmic/cytosolic categories for CDNs and EV/membrane categories for sEVs.

Spearman correlation across all replicates showed high within‐group reproducibility and clear separation among the three sample types (Figure 3B). Although the HEK293T cells and TurboID‐CDNs shared many protein identifications, their abundance profiles diverged broadly. Using a significance threshold of *fold change* ≥ 2 and two‐sided *p* < 0.05, a comparison identified 456 proteins that were enriched in TurboID‐CDNs and 729 enriched in the HEK293T cells within the TurboID‐HEK293T set (Figure 3C). For functional context, Gene Ontology (GO) Cellular Component enrichment (Figure 3D) was also performed on the proteins uniquely identified in TurboID‐CDNs (220 proteins, Figure 3A) and those that were significantly enriched in TurboID‐CDNs (456 proteins, Figure 3C). Analyses were conducted using DAVID 2021 (Dec 2021) and DAVID Knowledgebase v2023q4, revealing that the top terms were EV‐related. Conversely, the enrichment of nucleoplasm/nucleus terms emerged when analyzing the proteins unique to HEK293T cells (1161 proteins, Figure 3A) and those enriched in the same cells (729 proteins, Figure 3C). Moreover, a parallel comparison between the HEK293T cells and TurboID‐sEVs revealed widespread abundance shifts (Figure S3A), including enrichment of vesicle‐associated categories and depletion of nucleus/nucleoplasm terms (Figure S3B), consistent with efficient enrichment of vesicle proteins and removal of nuclear components during vesicle preparation.

Subsequently, a direct comparison was performed between TurboID‑CDNs and TurboID‑sEVs. As a reference‑only check, these vesicles were compared against a representative Top‑100 exosome marker panel curated from ExoCarta. The TurboID‐sEVs contained 89/100 markers, whereas the TurboID‐CDNs contained 82/100, indicating substantial, but incomplete, overlap with canonical exosome signatures (Figure S4). Pairwise identification counts identified 2019 proteins unique to TurboID‐CDNs, 221 unique to TurboID‐sEVs (Figure 3A), and 714 common to both. Within the common set, 137 were enriched in TurboID‐CDNs, and 266 were enriched in TurboID‐sEVs (Figure 3E). GO Cellular Component enrichment partitioned these differences, showing that sEV‐enriched proteins predominated in the EV and membrane categories, whereas CDN‐enriched proteins were linked to the mitochondrial, nucleoplasmic, and cytosolic components (Figure 3F). Collectively, these results demonstrate that cells and vesicles share extensive yet non‐identical proteomes. Indeed, compositional biases introduced by vesicle biogenesis (sEVs) versus fabrication (CDNs) yield distinct but overlapping landscapes.

### 2.3 Predicting Candidate Uptake‐Associated Routes From Vesicle Protein Composition

The differences in protein composition between TurboID‐CDNs and TurboID‐sEVs were subsequently examined to generate candidate predictions about uptake‐associated routes for each vesicle. Using curated route signatures (Table S1) for eight canonical modules—clathrin‐mediated endocytosis (CME) [41], clathrin‐independent CLIC/GEEC (CLIC) [42, 43], integrin‐assisted adhesion (INTE) [29, 30], heparan‐sulfate‐assisted uptake (HSPG) [44], scavenger/LDLR‐like uptake (SCAV) [45], caveolae‐mediated uptake (CAV) [46], macropinocytosis (MACRO) [47, 48], and phagocytic‐like uptake (PHAGO) [49, 50], route‐propensity scores (RPS) were computed from the vesicle‐side proteomes. “Anchors” were defined as vesicle‐surface determinants capable of directly mediating docking or adhesion to route‐defining receptors (e.g., integrins, selected adhesion molecules, and raft/capsule organizers on the outer leaflet). “Support” refers to vesicle‐surface co‐receptors and scaffolds (e.g., tetraspanins CD9/CD63/CD81, ICAM1, CD47, and flotillins) that modulate avidity or clustering but do not independently define uptake routes. “Synergy” tuples encode minimal co‐occurring pairs or triples (e.g., CAV1‐CAVIN1, LDLR‐LDLRAP1, and CDC42‐GRAF1) whose simultaneous detection strengthens route assignment beyond single‐gene matches. Recipient‐side cytosolic uptake machinery (e.g., AP‐2, clathrin, and dynamin) was excluded from vesicle‐side scoring. Scores were calculated using a surface‐biased subset (GO Cellular Component: cell surface/external side/plasma membrane; UniProt: signal peptide/transmembrane/GPI (glycosylphosphatidylinositol‐anchor)). “Anchors” were assigned greater weight than “Support” and “Synergy” (*w*_anchor_:*w*_support_:*w*_synergy_ = 0.6:0.3:0.1). Importantly, the qualitative conclusions remained robust during reasonable perturbations in these weights (Figure S5).

As shown in Figure 4A, TurboID‐CDNs exhibit broader and higher propensities across adhesion‐ and engulfment‐related routes than TurboID‐sEVs. The MACRO, INTE, and CLIC pathways are elevated in both vesicle types but are consistently higher in the CDNs, while CAV, SCAV, and PHAGO are detectable in the CDNs yet negligible in the sEVs. Similar CME levels were observed between the two vesicle types in this vesicle‐side readout, whereas the HSPG signals were weak. To illustrate predicted multi‐route compatibility for each vesicle, Figure 4B shows the per‐route RPS share (i.e., each RPS divided by the sum across all routes for that vesicle, ×100%). It can be seen that the CDN polygon expands across most axes, providing a composition‐derived prediction of broader route compatibility for CDNs than for sEVs. The source data for Figure 4 are provided in Table S4.

**FIGURE 4:**
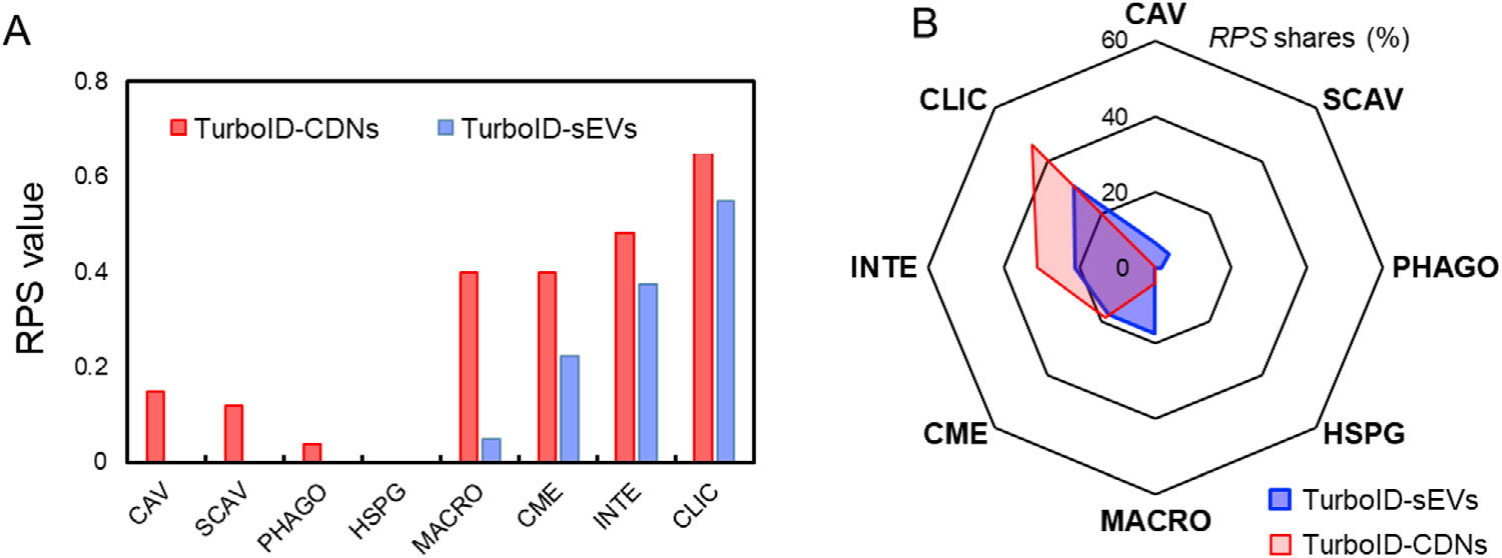
Uptake‑route propensity inferred from vesicle‑side proteomes.(A) RPS for eight uptake modules computed from surface‑biased vesicle proteomes. “Anchors” were assigned greater weight relative to “Support” and “Synergy.” (B) Radar plots showing the within‑vesicle RPS shares. The route signatures, mappings, and component counts can be found in Tables S1–S4. Figure S5 provides details regarding the robustness of the conclusions to weighting perturbations. RPS is a rule‐based hypothesis‐generating score and does not measure pathway flux or receptor dependence.

As presented in Figure 3, these composition‐derived propensities align with the global proteomics trends. Specifically, TurboID‐sEV proteins are enriched for membrane‐ and vesicle‐associated categories, thereby contributing to cellular homing. In contrast, TurboID‐CDN proteins include a broader array of cellular components. Consequently, the RPS analysis can be interpreted as a prior expectation that CDNs are predicted to be compatible with multiple routes. Notably, RPS is a rule‑based summary derived solely from vesicle‑side proteomes. CDN‐sEV differences should therefore be treated as hypothesis‐generating predictions for comparison with recipient‑side TurboID proximity interactomes.

### 2.4 Functional Activity of TurboID‐CDNs and Association With Recipient Cells

TurboID‐CDNs and control CDNs prepared from non‐transfected cells were incubated in vitro with ATP (1 mM), with or without biotin (50 µM), at 37°C for 4 h. Subsequently, the vesicles were precipitated, solubilized, and resolved by SDS‐PAGE (sodium dodecyl sulfate–polyacrylamide gel electrophoresis). Biotinylated proteins were detected using streptavidin–HRP, with the total protein serving as a loading control. TurboID‐negative and no‐biotin samples were processed in parallel; ATP was supplied during all tested reactions. The strongest ladder‐like streptavidin signal was observed for the TurboID‐positive, biotin‐containing condition, whereas the TurboID‐negative and no‐biotin controls showed lower signals (Figure S6A,C). Notably, the total protein profiles were comparable across all lanes (Figure S6B), supporting enzymatic labeling by vesicle‐associated TurboID under ATP‐supplemented conditions. Taken together, these results provide complementary evidence that the purified CDN preparation contains morphologically intact, enzymatically active TurboID‐associated vesicles. These assays do not determine the fraction of particles carrying the fusion protein, its copy number per particle, or its membrane topology; calibrated single‐particle measurements and fusion‐specific immunoblotting or protease‐protection analysis would be required to resolve these parameters.

Based on previous HEK293T uptake kinetics for sEVs [40, 51], recipient HEK293T cells were exposed to TurboID‑CDNs or TurboID‑sEVs for 4 h at 4.0  ×  10^10 ^particles/mL (as determined by nanoparticle tracking analysis) in the presence of biotin. Fluorescence imaging revealed punctate mCherry signals associated with recipient cells, consistent with binding and/or internalization (Figure 5A). A CCK‐8 concentration‐response assay performed with TurboID‐CDNs showed high recipient‐cell viability after 4 h, including at the working concentration (Figure 5B), supporting their implementation for proximity labeling.

**FIGURE 5:**
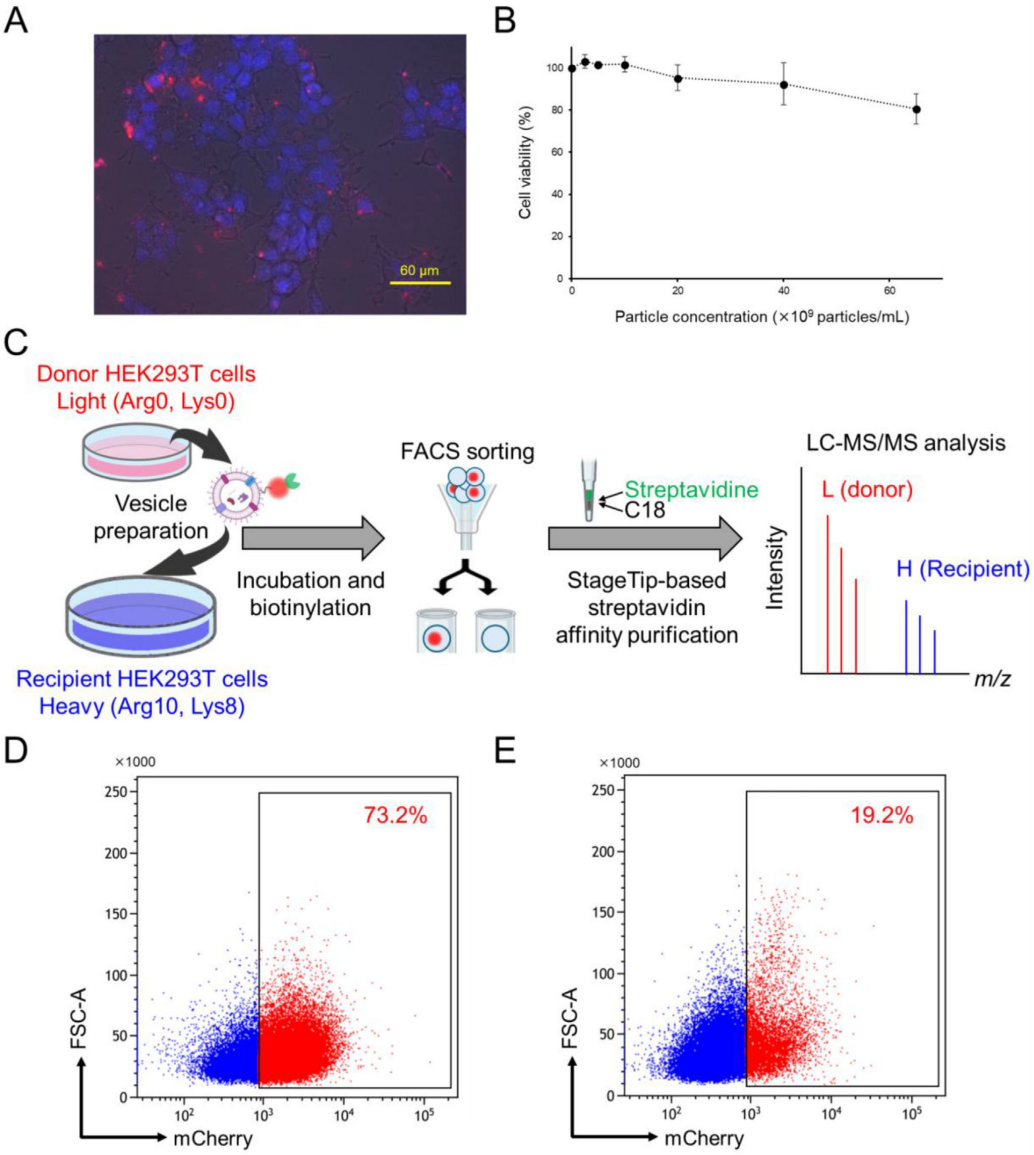
Association of TurboID‐CDNs and TurboID‐sEVs with recipient HEK293T cells for proximity labeling. (A) Representative epifluorescence image after 4 h TurboID‐CDN exposure (+ biotin); signal may be surface‐bound and/or internalized. (B) CCK‐8 viability after 4 h TurboID‐CDN exposure at the indicated concentrations. Data are presented as mean ± SD of three technical replicate wells (*n* = 3). (C) SILAC‐coded workflow and sorting. (D,E) Representative FACS plots for the CDN (D) and sEV (E) conditions, respectively, using the same gate defined by the no‐vesicle control. Percentages are events within the gate, not biological‐replicate statistics.

For downstream proteomics, the mCherry‑positive recipient cells were sorted using flow cytometry (Figure 5C). A single mCherry gate, defined on the negative control and maintained constant across samples, resolved a clear positive population for both vesicle types (Figure 5D,E). Because an acid‐ or protease‐based surface‐stripping step was not applied, the fixed gate identified total cell‐associated fluorescence and was used as an enrichment criterion rather than a quantitative measure of internalization. A clear mCherry‐positive population was recovered under each exposure condition, enabling matched downstream proximity‐proteomic processing; the displayed percentages should not be interpreted as relative internalization efficiencies because reporter loading per particle was not independently calibrated.

### 2.5 Recipient‑Side TurboID Proximity Labeling Identifies Candidate Uptake‐Associated Interactomes

To unambiguously discriminate the peptide origin, stable isotope labeling with amino acids in cell culture (SILAC) coding was employed (Figure 5C). Recipient HEK293T cells were labeled with heavy amino acids (L‐(^13^C_6_,^15^N_4_)‐arginine (Arg10) and L‐(^13^C_6_,^15^N_2_‐lysine (Lys8)), whereas the donor cells used to prepare the TurboID‑CDNs or TurboID‑sEVs were unlabeled (Arg0/Lys0). Under identical exposure conditions (4 h, +biotin 50 µM), the donor‑derived vesicle proteins were light, while the recipient‑derived proteins were heavy, enabling clear channel‑based assignment in the proximity datasets. mCherry‑positive recipient cells were isolated by fluorescence‑activated cell sorting (FACS) using a single gate defined on the negative control and maintained constant across samples. Additionally, mCherry‑negative fractions were processed in parallel as process‑matched controls. The enrichment of low‑abundance biotinylated species was performed using a StageTip‑based streptavidin workflow [40, 52, 53] prior to LC‑MS/MS analysis.

In the mCherry‐positive fractions, 839 heavy‐channel and 301 light‐channel proteins were identified following CDN exposure, whereas 803 heavy‐channel and 211 light‐channel proteins were identified following sEV exposure (Figure S7). The MFGE8(D49E)–mCherry–TurboID fusion protein was detected exclusively in the light channel in both datasets, providing an internal control for SILAC‐based discrimination between donor‐ and recipient‐derived proteins. Candidate recipient‐cell interactors were then operationally defined by comparing heavy‐channel intensities between the mCherry‐positive and mCherry‐negative fractions. Proteins with an mCherry‐positive/mCherry‐negative intensity ratio ≥ 1 were retained, yielding 432 candidates following CDN exposure and 700 following sEV exposure (Figure 6A,B). GO Cellular Component filtering further retained 183 and 289 membrane‐associated candidates, respectively. These proteins constitute a focused candidate set for molecular events associated with vesicle capture and/or internalization and are summarized in Table S5.

**FIGURE 6:**
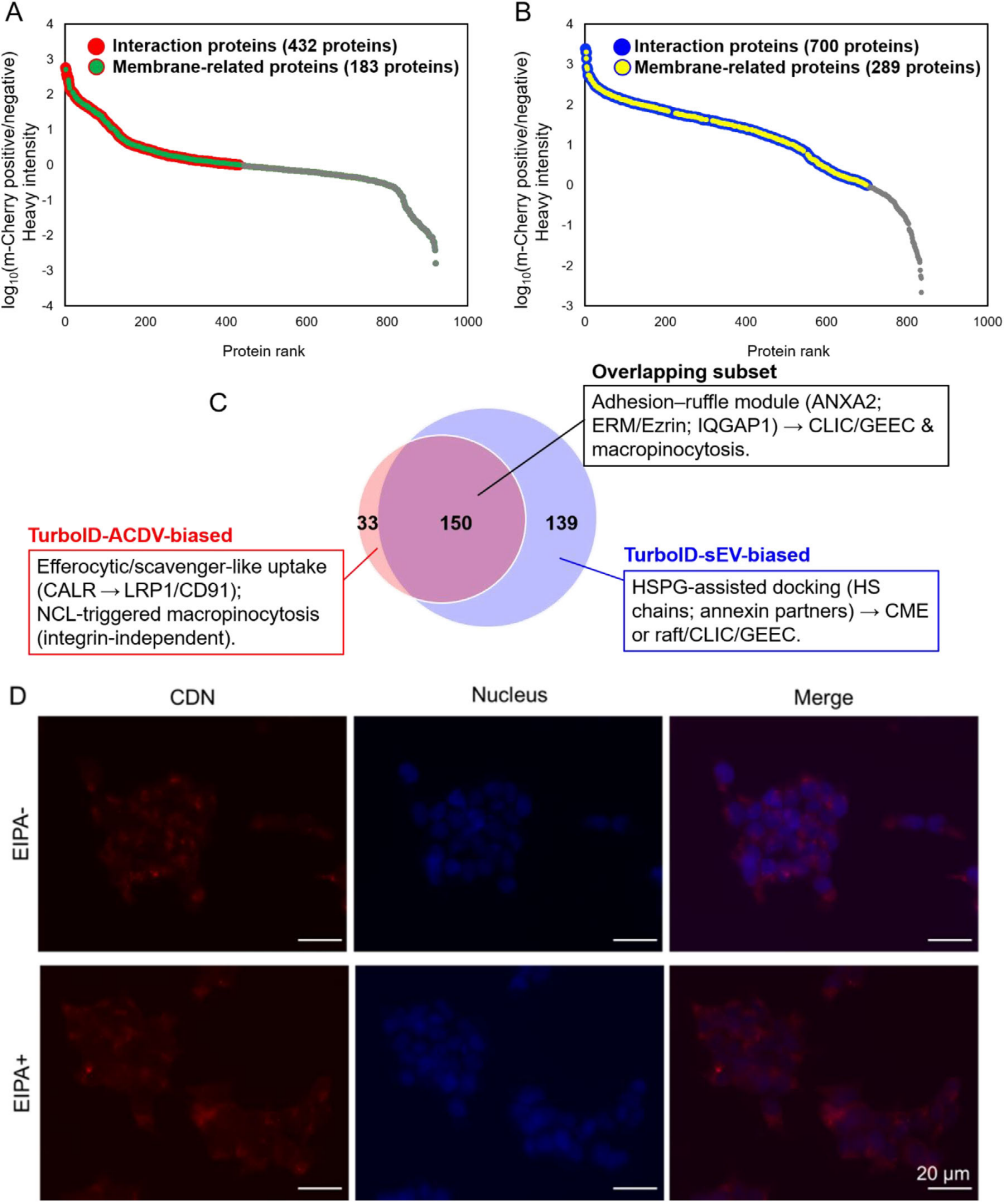
Recipient‐cell proximity datasets identify vesicle‐type‐biased candidate membrane associations.(A) Rank‐plot for the membrane proteins recovered from CDN‑exposed HEK293T cells (red; mCherry‑positive heavy channel relative to the mCherry‑negative control). The dots represent individual proteins, while the membrane proteins are highlighted in green. (B) Ranked enrichment plot for the corresponding sEV‑exposed condition (blue), wherein the membrane proteins are highlighted in yellow. (C) Venn diagram summarizing the membrane proteins retained by GO Cellular Component filtering. The overlapping subset is enriched for an adhesion–ruffle module, while TurboID‐CDN‑specific proteins include CALR and NCL, and sEV‑specific proteins correspond to HSPG‑assisted capture upstream of clathrin‑mediated or CLIC/GEEC internalization. (D) Representative fluorescence images of cell‐associated CDN signals in recipient HEK293T cells with or without EIPA treatment.

Recipient‐side proximity labeling resolved distinct condition‐associated membrane protein cohorts (Figure 6C), which were evaluated together with the composition‐derived route‐propensity predictions shown in Figure 4. The CDN‐associated cohort contained recipient‐derived CALR and NCL. Given the established links between CALR–LRP1/CD91 interactions and efferocytic recognition and between cell‐surface NCL and macropinocytic processes, these observations nominate LRP1/CD91‐linked efferocytic recognition and NCL‐associated macropinocytic processes as candidate mechanisms for CDN–cell engagement [54, 55].

By contrast, the sEV‐associated cohort contained proteins consistent with an HSPG‐associated docking model and clathrin‐ or CLIC/GEEC‐related membrane organization, in agreement with the route‐propensity profile shown in Figure 4. For example, ANXA2 and related annexin‐family proteins have been reported in HS‐associated adhesion contexts. The subset shared between the CDN and sEV conditions included annexins and ERM—family linkers that connect cortical actin to PtdIns(4,5)P2‐rich microdomains, supporting a common adhesion–ruffle module. Similar membrane organization has been linked to CLIC/GEEC‐like endocytosis and macropinocytic ruffle capture of nanoscale cargos [56, 57, 58]. Together, these condition‐associated cohorts nominate distinct and partially shared mechanistic models for vesicle–cell engagement. Notably, they do not establish a causal uptake route or a relative internalization efficiency on their own.

To functionally probe the contribution of the macropinocytic pathways implied by the proximity interactome, CDN uptake was assessed under pharmacological inhibition conditions. Specifically, recipient HEK293T cells were pretreated with 5‐(N‐ethyl‐N‐isopropyl)‐amiloride (EIPA, 30 µM, 30 min) to suppress macropinocytosis, followed by incubation for 4 h with fluorescently labeled CDNs. Fluorescence microscopy revealed comparable intracellular fluorescence intensities between inhibitor‐treated and untreated cells (Figure 6D), indicating that CDN internalization was largely unaffected by EIPA under these conditions. This result suggests that CDNs are internalized through multiple, partially compensatory routes. The inhibition of macropinocytosis may therefore up‐regulate alternative entry pathways, consistent with the multi‐route propensity predicted from their proteomic composition.

Several limitations define the scope of the mechanistic interpretation. First, conventional washing and mCherry‐based sorting measure total cell association and do not distinguish surface‐bound from internalized vesicles. Second, the single 4 h endpoint combines initial docking with any subsequent trafficking events. Third, proximity enrichment and rule‐based route‐propensity scoring identify candidate associations but do not establish receptor necessity, pathway sufficiency, or the fraction of vesicles entering through a given route. Finally, cell‐associated fluorescence may be influenced by differences in reporter abundance per particle. Surface stripping, 4°C versus 37°C and time‐course controls, quantitative organelle colocalization, competitive ligands, and receptor‐ or pathway‐specific genetic perturbations will be required to resolve these questions.

Together, these findings support the use of dual‐sided proteomics to prioritize candidate vesicle–cell interactions for subsequent functional testing. Future investigations should combine surface engineering with quantitative internalization assays, receptor‐specific perturbations, and time‐resolved proximity labeling or colocalization to determine how individual routes contribute to delivery.

## 3 Conclusions

This study establishes a dual‐sided proteomics framework that links vesicle composition to the molecular interface formed with recipient cells. Although extrusion‐generated CDNs and sEVs shared similar bulk physicochemical properties, they exhibited markedly distinct proteomic architectures and recipient‐cell proximity landscapes. CDNs displayed a broader repertoire of predicted uptake‐associated signatures, and the CDN‐associated recipient‐cell cohort contained CALR and NCL, nominating LRP1/CD91‐linked efferocytic recognition and NCL‐associated macropinocytic processes. By contrast, the sEV‐associated cohort contained signatures consistent with HSPG‐assisted docking and clathrin‐ or CLIC/GEEC‐related uptake. Together, these complementary datasets reveal that mechanically generated CDNs engage recipient cells through a molecular landscape distinct from that of naturally secreted sEVs and identify specific receptor‐ and pathway‐level candidates for functional validation. Although the present design does not directly establish receptor dependence or distinguish surface association from internalization, it resolves vesicle‐type‐associated molecular differences and focuses subsequent experiments on defined mechanisms. By integrating vesicle‐side composition with recipient‐side proximity labeling, this approach provides a powerful framework for dissecting vesicle–cell communication and guiding the rational engineering of cell‐derived nanocarriers with more predictable cell‐interaction profiles.

## 4 Experimental Section

### 4.1 Cloning

The entry clone pENTR221 MFGE8 for Gateway cloning was purchased from DNAFORM (Japan). Site‐directed mutagenesis was performed to generate the MFGE8 D49E mutant using the QuikChange Lightning site‐directed mutagenesis kit (Agilent, USA) with the following primers: forward, 5′‐GTACGAGGGGAAGACCTCTCCTCGCACTTCT‐3′, and reverse, 5′‐AGAAGTGCGAGGAGAGGTCTTCCCCTCGTAC‐3′. Subsequently, the MFGE8 D49E coding sequence was subcloned into the destination vector pDEST‐pcDNA5, containing mCherry‐TurboID, using LR Clonase II (Thermo Fisher Scientific, USA) according to the Gateway cloning protocol. All coding regions constructed in this study were verified by Sanger dideoxy sequencing.

### 4.2 Cell Culture

HEK293T cells (obtained from RIKEN BRC, Japan) were maintained in Dulbecco's Modified Eagle Medium (DMEM; Thermo Fisher Scientific) supplemented with 10% fetal bovine serum (FBS; Thermo Fisher Scientific) at 37°C in a humidified incubator with 5% CO2. SILAC labeling was achieved by culturing the cells for at least five doubling times in arginine‐ and lysine‐free DMEM (Thermo Fisher Scientific) supplemented with 10% sEV‐depleted FBS and heavy isotope‐labeled amino acids [0.398 mM L‐(^13^C_6_, ^15^N_4_)‐arginine (Arg10) and 0.798 mM L‐(^13^C_6_, ^15^N_2_‐lysine (Lys8)) surface charge; Cambridge Isotope Laboratories, USA]. Additionally, sEV‐depleted FBS was prepared by the ultracentrifugation of standard FBS at 100 000 × g for 18.5 h, and the resulting supernatant was used as sEV‐depleted FBS.

### 4.3 Plasmid Transfection

On the day of transfection, HEK293T cells were cultured in 15 cm dishes until reaching ∼50% confluency. The cells were then transfected with a mixture containing MFGE8 D49E‐mCherry‐TurboID plasmid DNA (15 µg) and 1.0 mg/mL polyethylenimine (45 µL; PEI; Polysciences, USA). Following transfection, the cells were incubated for 24 h at 37°C in a humidified incubator with 5% CO_2_.

### 4.4 Preparation of the TurboID‐CDNs

TurboID‐expressing HEK293T cells were detached from culture dishes using a cell scraper, transferred into centrifuge tubes, and pelleted by centrifugation. The supernatant was discarded, and the cells were resuspended in PBS. The cell concentration was determined using 0.4% trypan blue staining. After transferring the required number of cells to a 15 mL tube, the cells were centrifuged, the supernatant was removed, and the pellet was washed once again with PBS. The cells were then resuspended in HEPES/KOH buffer at a concentration of 1 × 10^7^ cells/mL, aliquoted into 1.5 mL low‐binding microtubes (maximum 750 µL per tube), and subjected to three freeze‐thaw cycles (freezing at −80°C and thawing at room temperature). Following this, the samples were centrifuged at 4000 × g and 4°C for 3 min, and the wash procedure was repeated three times to remove soluble proteins. The resulting pellet was resuspended in HEPES/KOH buffer at a concentration of 1 × 10^7^ cells/mL, dispersed by bath sonication (28 kHz, 1–4 min, ice bath), and then sequentially extruded through polycarbonate membranes (11 passes through a 0.4 µm filter, followed by 21 passes through a 0.2 µm filter) using a Mini‐Extruder (Avanti Polar Lipids, USA). To reduce unbound and loosely associated soluble proteins, the TurboID‐CDNs were transferred to a centrifugal ultrafiltration spin column with a molecular weight cutoff of 300 kDa (pre‐washed with HEPES buffer) and centrifuged at 14 000 × g and 4°C. The retentate was washed once more by adding an equal volume of fresh HEPES/KOH buffer and centrifuging under the same conditions. Finally, the purified TurboID‐CDNs were resuspended and recovered in HEPES/KOH buffer.

### 4.5 Ultracentrifugation for TurboID‐sEV Isolation

The procedure for sEV isolation via ultracentrifugation was adapted with slight modifications from a standard protocol commonly used in sEV research. Specifically, cell‐conditioned medium was collected from TurboID‐expressing HEK293T cells cultured in 15 cm dishes at ∼80% confluency. All subsequent centrifugation steps were performed at 4°C. Initially, cells were removed by centrifugation at 400 × g for 10 min, and the cell debris was further cleared by centrifugation at 2000 × g for 10 min. To pellet and remove any large EVs, the supernatant was subjected to ultracentrifugation at 15 000 × g for 40 min. The resulting supernatant was filtered through a 0.22 µm polyethersulfone membrane filter (Merck, Germany). The filtered samples were then subjected to ultracentrifugation at 100 000 × g for 120 min. The obtained pellets were washed with ice‐cold PBS and subjected to an additional round of ultracentrifugation under identical conditions (100 000 × g for 120 min) to yield the purified TurboID‐sEVs.

### 4.6 Zeta Potential Measurement

Zeta potential measurements were carried out in 10 mM PBS at 25°C using a Zetasizer Nano ZSP instrument (Malvern Instruments, UK). Three independent experiments were performed. CDNs and sEVs from wild‐type and TurboID‐expressing HEK293T cells were diluted to 1 µg protein/mL for zeta potential measurements.

### 4.7 Nanoparticle Tracking Analysis

The concentrations and size distributions of the CDNs and sEVs were measured based on Brownian motion using a NanoSight NS300 instrument (Malvern PANalytical, UK). For each sample, five individual 60‐s acquisitions were performed, and the mean values from these measurements were used to determine the mode of the particle size distribution and the nanoparticle concentration.

### 4.8 TurboID‐Reaction In Vitro

Pelleted TurboID‐CDNs were resuspended in PBS (1 mL) and biotinylation was initiated by adding biotin (final concentration = 50 µM) and ATP (final concentration = 1 mM; adenosine 5'‐triphosphate disodium salt trihydrate; Fujifilm Wako, Japan) dissolved in 1 M Tris‐HCl (pH 7.5), followed by incubation at 37°C for 4 h. After this time, any free biotin was removed and the proteins were precipitated by the addition of ice‐cold acetone to the suspension (final concentration = 80%). The resulting samples were incubated at −20°C for 1 h, then subjected to centrifugation at 20 000 × g and 4°C for 10 min. The supernatant was carefully removed, and the pellet was gently washed once with ice‐cold acetone. Any residual acetone was evaporated using a centrifugal vacuum concentrator, and the resulting protein pellet was resuspended in RIPA buffer (100 µL).

### 4.9 Immunoblotting

LDS (lithium dodecyl sulfate) buffer (pH 8.4, Thermo Fisher Scientific) containing 50 mM dithiothreitol (DTT, Fujifilm Wako) was added to the TurboID‐CDN protein preparation in RIPA buffer, followed by heating at 70°C for 5 min. The samples were separated by SDS‐PAGE (e‐PAGEL 12%; ATTO Corporation, Japan), after which the proteins were transferred onto polyvinylidene fluoride membranes. These membranes were then blocked overnight with gentle mixing in Bullet Blocking One for Western Blotting (Nacalai Tesque, Japan). Subsequently, the membranes were incubated with streptavidin–HRP (diluted 1:2000) for 1 h. After washing four times with TBS‐t, the membranes were incubated with ECL Western blotting substrate (GE Healthcare, USA) for signal detection.

### 4.10 Transmission Electron Microscopy

The CDNs were applied to an ion‐sputter plasma‐treated elastic carbon grid for 10 min, then washed and negatively stained for 30 min using an EM stainer (NisshinEM, Japan), diluted 3‐fold in 50 mM HEPES/KOH buffer (pH 7.4). The stain was then removed using filter paper, and the grid was dried. The sEV samples were observed using an HT7700 transmission electron microscope (Hitachi, Japan).

### 4.11 Single‐Particle Analysis Using Imaging Flow Cytometry

The protein concentration of TurboID‐CDNs was determined using the Pierce BCA Protein Assay Kit (Thermo Fisher Scientific). Initially, the vesicles were labeled with the ExoSparkler Exosome Membrane Labeling Kit–Deep Red (DOJINDO, Japan) according to the manufacturer's instructions. Labeled vesicles were collected in 50 mM HEPES/KOH buffer (50 µL, pH 7.4) and diluted 1:1000 with the same buffer prior to analysis. Imaging flow cytometry was performed using an AMNIS ImageStreamX Mark II instrument (Cytek Biosciences, USA). Prior to each measurement, calibration was conducted using SpeedBeads (Cytek Biosciences, USA). The flow velocity was adjusted based on the mean bead velocity in the channel, while the side scatter intensity was calibrated using the default signal of SpeedBeads under 785 nm laser illumination. The laser outputs were calibrated using the scatter intensity of flowing SpeedBeads. Bright‐field images were collected in channel 1 (Ch1), TurboID‐fused proteins were detected in Ch3, ExoSparkler‐Deep Red fluorescence was detected in Ch5, and side scatter (SSC) was detected in Ch6. The laser powers were set to 200 mW for 561 nm, 150 mW for 642 nm, and 70 mW for SSC. All measurements were performed under low‐flow conditions using a 60 × objective lens. For the detergent disruption tests, Triton X‐100 (Nacalai Tesque, Japan) was added to the TurboID‐CDNs at a final concentration of 0.1%, and particle counts were recorded under identical conditions. The number of events was recorded per minute for each sample.

### 4.12 TurboID Reaction in Recipient Cells

TurboID‐CDNs and TurboID‐sEVs were prepared at a final concentration of 4 × 10^10^ particles/mL in DMEM containing SILAC heavy amino acids and 10% sEV‐depleted FBS, and then added to recipient HEK293T cells previously labeled with SILAC heavy amino acids. Biotin was simultaneously added to give a final concentration of 50 µM, and the cells were incubated at 37°C for 4 h. After incubation, the cells were harvested and resuspended in PBS at a density of ∼2.0 × 10^6^ cells/mL. The mCherry‐positive and mCherry‐negative cell populations were sorted using the cell‐sorting procedure detailed below. The sorted cells were lysed, and the biotinylated proteins were enriched using C18‐streptavidin Sepharose SpinTips (Cytiva, USA), as described later.

### 4.13 Fluorescence‑Activated Cell Sorting (FACS)

Following the TurboID reaction, the cells were collected and washed three times by centrifugation at 1000 × g and 4°C for 3 min. The washed cells were then resuspended in BD Pharmingen staining buffer (BD Biosciences, USA), and the mCherry‐positive and mCherry‐negative populations were separated using an MA900 Cell Sorter (Sony, Japan). Gating thresholds were defined based on the no‐vesicle control and applied identically across all samples. Serum‐free SILAC medium was used as the collection buffer during sorting to minimize contamination. The sorted fractions were centrifuged at 1000 rpm and room temperature for 10 min, washed twice with PBS under the same conditions, resuspended in PBS, and stored at −80°C until required for proteomic analysis. The sorted cells were then lysed, and biotinylated proteins were enriched using a C18‐streptavidin StageTip, as described in a previous study.40 The cell viability of the recipient HEK293T cells after TurboID‐CDN exposure was evaluated using Cell Counting Kit‐8 (DOJINDO LABORATORIES, Japan) according to the manufacturer's instructions.

### 4.14 Protein Digestion and Purification of Peptide Samples for LC‐MS/MS Analysis

The CDNs and sEVs were digested using the phase‐transfer surfactant (PTS)‐aided trypsin digestion protocol, as described previously [59]. Specifically, the samples were suspended in PTS buffer (200 µL; 12 mM sodium deoxycholate, 12 mM sodium lauroyl sarcosinate in 100 mM Tris‐HCl, pH 9.0) containing protease inhibitors. The samples were incubated on a heating block at 95°C for 5 min and then sonicated for 20 min. The extracted proteins were quantified using a BCA protein assay kit, and reduced with 10 mM DTT for 30 min at 37°C, followed by alkylation with 50 mM 2‐iodoacetamide (IAA; FUJIFILM Wako) for 30 min at room temperature in the dark. After this time, the samples were diluted 5‐fold with 50 mM ammonium bicarbonate buffer, and the proteins were digested using lysyl endopeptidase (LysC) (FUJIFILM Wako) and trypsin (Promega, Japan) overnight at 37°C in a shaking incubator. Subsequently, ethyl acetate (1 mL) was added to an aliquot (1 mL) of the digested solution, and the mixture was acidified using 0.5% trifluoroacetic acid (TFA, final concentration). The samples were then vortexed for 2 min and centrifuged at 15 800 × g for 2 min to separate the aqueous and organic phases. The aqueous phase was collected, dried, and the resulting solid was resuspended in a mixture of 0.1% TFA in 5% acetonitrile (ACN) and loaded onto a StageTip containing SDB‐XC (upper) and SCX (bottom) Empore disk membranes (GL Sciences, Japan) [53, 60, 61]. The peptides were then washed with 0.1% TFA in 5% ACN and 0.1% TFA in 80% ACN. Subsequently, the peptides were eluted from the StageTip using 500 mM ammonium acetate containing 4% TFA and 30% ACN, then 500 mM ammonium acetate containing 30% ACN. The sample was dried and resuspended using 0.5% TFA in 5% ACN. Finally, the peptides were desalted once more using a StageTip equipped with an SDB‐XC Empore disk membrane, and suspended in loading buffer (0.5% TFA in 5% ACN) for subsequent LC‐MS/MS analyses. The peptide concentration was also measured using a NanoDrop spectrophotometer (Thermo Fisher Scientific) at 205 nm, applying an extinction coefficient of 31 [62].

### 4.15 SpinTip‐Based Streptavidin Affinity Purification

The biotinylated proteins were purified using streptavidin Sepharose as described previously [40, 52]. Specifically, two plugs of C18 solid‐phase extraction discs (GL Sciences, Japan) were placed in a 200 µL pipette tip. The tips were conditioned by adding methanol (60 µL) and centrifuging at 1000 × g for 1 min, followed by blocking of the C18 discs with 2% SDS (60 µL) at 1500 × g for 1 min. Subsequently, an aliquot (1 µL) of the streptavidin Sepharose slurry was loaded onto each tip and briefly centrifuged at 600 × g for 1 s. The extracted proteins were then loaded onto the prepared tips and subjected to centrifugation at 100 × g for 1 h to capture the biotinylated proteins. After capture, the tips were washed four times with RIPA buffer (60 µL) at 1500 × g for 1 min per wash. For peptide digestion, the C18 discs were activated with 0.5% (v/v) acetic acid in 80% ACN (60 µL) at 1500 × g for 1 min. Cysteine residues were reduced by adding 50 mM ammonium bicarbonate buffer containing 10 mM DTT (20 µL), followed by incubation at room temperature for 15 min and removal of the solution by centrifugation at 1× g for 30 s. Subsequently, an aliquot (2 µL) of digestion buffer containing 0.25 µg/µL trypsin, 50 mM IAA, and 50 mM ammonium bicarbonate buffer was loaded onto each tip, and the samples were incubated at 37°C for 1 h to achieve simultaneous protein digestion and alkylation. The digested peptides retained on the C18 discs were washed with 1% formic acid (60 µL) at 1500 × g for 1 min and subsequently eluted with 0.5% (v/v) acetic acid in 80% ACN (60 µL) at 200 × g for 5 min. The peptides were further purified using SDB‐XC‐SCX and SDB‐XC StageTips, as described above.

### 4.16 LC‐MS/MS Analysis

LC‐MS/MS analysis was carried out in data‐dependent acquisition (DDA) mode with a FAIMS Pro Duo interface connected to an Orbitrap Fusion Lumos Tribrid MS. The electrospray voltage was set to 2.4 kV in the positive ion mode. The FAIMS mode was set to standard resolution, and the total carrier gas flow was 4.6 L/min. The compensation voltage (CV) was set to −40, −60, or −80 V, and the cycle time for each CV experiment was 1 s. The mass range of the survey scan ranged from 300 to 1500 m/z with a resolution of 120 000, standard automatic gain control (AGC), and a maximum injection time of 50 ms. The MS/MS scan was performed using an ion trap with a rapid ion trap scan rate, standard AGC, a maximum injection time of 35 ms, and an isolation window of 1.6 m/z. The precursor ions were fragmented by higher‐energy collisional dissociation using a normalized collision energy of 30%. The exclusion duration was set to 20 s. LC was performed using an Ultimate 3000 pump (Thermo Fisher Scientific) and an HTC‐PAL autosampler (CTC Analytics, Switzerland) equipped with self‐pulled needle columns (150 mm length, 100 µm ID, 6 µm needle opening) packed with Reprosil‐Pur 120 C18‐AQ 1.9 µm reversed‐phase material (Dr. Maisch, Ammerbuch, Germany) [63]. The injection volume was 5 µL, and the flow rate was 500 nL/min. Separation was achieved by applying a three‐step linear gradient of 4%–8% ACN (in 0.5% acetic acid) for 5 min, 8–32% ACN for 60 min, 32–80% ACN for 5 min, and 80% ACN for 10 min.

### 4.17 Proteomics Data Processing and Analysis

All raw MS data files were processed and analyzed using MaxQuant (version 1.6.17.0) [64], with database searches performed using the integrated Andromeda search engine [64, 65] against the human UniProt FASTA database (release 2019‐3), supplemented with common contaminants and enzyme sequences. The raw data files obtained from the FAIMS experiments were initially converted into MaxQuant‐compatible MzXML files using FAIMS MzXML Generator (https://github.com/coongroup/FAIMS‐MzXML‐Generator) [66]. Search parameters allowed up to two missed cleavages and included variable modifications: methionine oxidation, protein N‐terminal acetylation, and, for the SILAC experiments, incorporation of heavy amino acids (Arg10 and Lys8). The carbamidomethylation of cysteine was specified as a fixed modification. Mass tolerances were set to 4.5 ppm for the precursor ions and 20 ppm for the MS/MS fragments. The false discovery rate was controlled at 1% for both the peptide‐spectrum match and protein levels. Protein‐level quantification was performed across all experiments using ‘unique plus razor’ peptides, and proteins quantified by at least one unique peptide were retained. To generate the volcano plots, only proteins quantified in at least two of three replicates under at least one experimental condition were included in the subsequent analysis. When multiple accessions were assigned to a protein group, the first accession listed in the Protein IDs column was used as the representative identifier for downstream annotation, mapping, and plotting. For interactome analysis, missing values were imputed as half of the minimum intensity observed for the quantified protein within the same dataset to evaluate the relative abundance.

### 4.18 GO Enrichment

GO enrichment was performed in DAVID (v2021) and DAVID Knowledgebase (v2023q4) [67, 68]. Protein identifiers were supplied as UniProt accessions, with isoform suffixes collapsed to the parent entry. The statistical universe comprised the union of all UniProt accessions identified in any sample in this study (‘All IDs’ background). Enrichment *P*‑values were computed using DAVID's modified Fisher's exact test; unless otherwise noted, Benjamini–Hochberg‐adjusted *p*‐values are reported.

### 4.19 Route‑Propensity Analysis From Vesicle Proteomes

Proteins identified in the comparative proteomics were converted to UniProt accessions with isoform suffixes removed. Three lists were generated, namely CDN‑specific, sEV‑specific, and Shared (overlap). The effective sets used for scoring were defined as CDN_eff_ = CDN‐specific ∪ Shared and sEV_eff_ = sEV‐specific ∪ Shared. The union of all CDN and sEV identifications served as the background universe from which all route signatures were derived.

Route signatures: For each uptake route (i.e., CME, CLIC, INTE, HSPG, SCAV, CAV, MACRO, and PHAGO), a compact signature was curated, and partitioned into “Anchors”, “Support”, and “Synergy” tuples (Table S1). Evidence strings referenced route‑level canonical reviews (CME [41], CLIC [42, 43], INTE [29, 30], HSPG [44], SCAV [45], CAV [46], MACRO [47, 48], and PHAGO [49, 50]) and, where applicable, included family‑based heuristics (e.g., integrins, scavenger receptors) and Synergy tuples (e.g., CAV1+CAVIN1). UniProt‑to‑HGNC mapping was performed using org.Hs.eg.db with the UniProt REST API (organism_id = 9606, accessed 2025‑08) as fallback. The full mapping table is provided in Table S2. To focus on vesicle‑surface determinants, GO Cellular Component annotations (cell surface, external side of plasma membrane, plasma membrane, and extracellular region) were combined with UniProt topology information (signal peptide, transmembrane, GPI) to generate a discrete SurfaceScore (0–4); proteins with *SurfaceScore* ≥ 2 were retained for RPS calculation (Table S3). RPS was determined based on the presence or absence of each protein in the filtered set, without the use of quantitative abundance values.

**Definition of the route‑propensity score**: For a given vesicle set *G*(CDN_eff_ or sEV_eff_) and route definition *R_r_* = (*A_r_*,*S_r_*,*T_r_*) with Anchors *A_r_*, Support *S_r_*, and Synergy tuples *T_r_* = {*t*_1_,…,*t*_k_}, we defined the route‑propensity score

$$\begin{array}{cc} RPSr\left(G\right) & =w_{anchor}*\frac{\left|G\cap Ar\right|}{\left|Ar\right|}+w_{support}*\frac{\left|G\cap Sr\right|}{max\left(1,\;\left|Sr\right|\right)}\\ & \;+w_{synergy}*\frac{1}{max\left(1,\;\left|Tr\right|\right)}\sum_{t\in Tr}1\left[t\subseteq G\right] \end{array}$$

where ∣·∣ denotes set cardinality and 1[𝑡⊆𝐺] is an indicator function that equals 1 when all proteins in the tuple 𝑡 are present in *G*, and 0 for all other cases. If 𝑇𝑟 is empty, the Synergy term is defined as 0 based on the max(1,∣𝑇𝑟∣) denominator. By construction, each term ranges from 0 to 1, ensuring that the overall score satisfies 0 ≤ *RPS*𝑟(𝐺) ≤ 1. Throughout Figure 4, the following weighting was employed: 𝑤_anchor_:𝑤_support_:𝑤_synergy_ = 0.6:0.3:0.1. Alternative weight triplets (i.e., 0.7:0.2:0.1 and 0.5:0.4:0.1) yielded qualitatively similar route rankings and CDN‐sEV contrasts (Figure S5). Scores were calculated independently for the TurboID‑CDNs and TurboID‑sEVs using the same route signatures and background universe. The per‑route matched/total counts for Anchors and Support, along with the fraction of satisfied Synergy tuples, are summarized together with the RPS values in Table S4.

### 4.20 Exploratory EIPA Treatment

HEK293T cells were seeded in 6‐well plates at 4.0 × 10^5^ cells/well and cultured for 24 h. The cells were pretreated with 30 µM EIPA (Cayman Chemical, USA) for 30 min, followed by incubation for 4 h with fluorescently labeled TurboID‐CDNs (4.0 × 10^10^ particles/mL), which were prepared using the ExoSparkler Exosome Membrane Labeling Kit–Deep Red (DOJINDO, Japan). After incubation, the cells were washed with PBS and observed by IX71 fluorescence microscopy (Olympus, Japan).

## Author Contributions

Eisuke Kanao, Koshi Imami and Yoshihiro Sasaki conceived this work. Eisuke Kanao, Ryosuke Mizuta, Saki Tarao, Yukika Hara, and Yuka Li performed the cell culture and proteomic experiments. Saki Tarao performed the FACS experiments. Eisuke Kanao, Ryosuke Mizuta, Yasushi Ishihama and Yoshihiro Sasaki analyzed the data. Eisuke Kanao, Ryosuke Mizuta, and Yoshihiro Sasaki wrote the original manuscript. Eisuke Kanao, Ryosuke Mizuta, Kazunari Akiyoshi, Yasushi Ishihama, Koshi Imami, and Yoshihiro Sasaki discussed the results and contributed to the manuscript.

## Conflicts of Interest

The authors declare no conflicts of interest.

## Supporting information




**Supporting File**: smtd71014‐sup‐0001‐SuppMat.docx.

**Supporting File**: smtd71014‐sup‐0002‐Sup.zip.

## Data Availability

All processed data are provided in Tables S1–S5. The mass spectrometry proteomics data have been deposited with the ProteomeXchange Consortium via the jPOST partner repository (https://jpostdb.org) [69] under the dataset identifier PXD071934.
