# Supplementary material for: Proximity Proteomics Maps Candidate Cellular Uptake Pathways for Cell‐Derived Nanovesicles
Source: Small Methods. 2026 Sep 2;10(19):e71014. doi: 10.1002/smtd.71014 (PMC13646036; doi:10.1002/smtd.71014)
Supplement: Supplementary file 1 [file SMTD-10-e71014-s002.docx]

**Supporting Information**

**Proximity Proteomics Maps Candidate Cellular Uptake Pathways for Cell-Derived Nanovesicles**

Eisuke Kanao,^1,2#,*^ Ryosuke Mizuta,^3#^ Saki Tarao,^3^ Yukika Hara,^3^ Yuka Li,^1^ Kazunari Akiyoshi,^4^ Yasushi Ishihama,^1,2^ Koshi Imami,^5^ Yoshihiro Sasaki^3,*^

1) Graduate School of Pharmaceutical Sciences, Kyoto University, Kyoto 606–8501, Japan

2) Laboratory of Proteomics for Drug Discovery, National Institute of Biomedical Innovation, Health and Nutrition, Ibaraki, Osaka 567-0085, Japan.

3) Graduate School of Engineering, Kyoto University, Kyoto 606–8501, Japan

4) Graduate School of Medicine, Kyoto University, Kyoto 606–8501, Japan

5) Proteome Homeostasis Research Unit, RIKEN Center for Integrative Medical Sciences, 1-7-22 Suehiro-cho, Tsurumi-ku, Yokohama, Kanagawa 230-0045, Japan

^#^These two authors equally contributed.

*Correspondence and requests for materials should be addressed to EK and YS.

Eisuke Kanao: kanao.eisuke.7s@kyoto-u.ac.jp

Yoshihiro Sasaki: sasaki.yoshihiro.8s@kyoto-u.ac.jp

Keywords: Cell-derived nanovesicle, Extracellular vesicle, Cellular Uptake Pathway, Proximity labeling

**Figure S1.** Size distribution and morphology of TurboID‑CDNs and sEVs.

**Figure S2.** Abundance‑rank distributions (iBaq‑based) and TurboID fusion ranking.

**Figure S3.** Comparison of the proteome profile between TurboID‑sEVs versus the parental cells.

**Figure S4.** Coverage of canonical sEV markers by TurboID‑CDNs and TurboID‑sEVs.

**Figure S5.** Weight-sensitivity analysis for RPS.

**Figure S6.** *In‑vitro* biotinylation activity of TurboID-CDNs.

**Figure S7.** Venn diagrams summarizing identifications in heavy (recipient) and light (donor) channels for mCherry‑positive cells.

**Figure S1.** Size distribution and morphology of TurboID‑CDNs and sEVs.

NTA profiles for TurboID‑CDNs (A), wild‑type HEK293T‑derived CDNs (B), and sEVs (C). Representative TEM micrographs (right panels in A and B) confirm spherical morphology for both CDN preparations.

**Figure S2. Abundance‑rank distributions (iBaq‑based) and TurboID fusion ranking.**

Abundance‑rank curves derived from iBaq values for (A) TurboID-HEK293T cells, (B) TurboID‑CDN, and (C) TurboID‑sEVs. The TurboID fusion is highlighted and ranks within the top ~3% of each distribution.

**Figure S3.** **Comparison of the proteome profile between TurboID‑sEVs versus the parental cells.**

(A) Volcano plot comparing TurboID‑sEVs with the parental cells. Points to the right (left) correspond to proteins with higher abundance in TurboID‑sEVs (parental cells). (B) Gene Ontology (cellular component) enrichment. Top: union of proteins uniquely identified in sEVs and proteins showing higher abundance in sEVs versus the parental cells. Bottom: union of proteins uniquely identified in the parental cells and proteins showing higher abundance in the cells versus sEVs.

**Figure S4. Coverage of canonical sEV markers by TurboID‑CDNs and TurboID‑sEVs.**

Presence/absence matrix for a representative Top‑100 sEV marker set (ExoCarta). Colors denote detection in both vesicle types (light green), TurboID‑CDNs only (blue), TurboID‑sEVs only (orange), or not detected (gray). As a reference‑only check, we compared our vesicles against a representative Top‑100 exosome marker panel curated from ExoCarta (query date: 2025-8). This panel serves as an illustrative benchmark rather than a definitive ground truth.

**Figure S5. Weight-sensitivity analysis for *RPS*.**

Bar plots comparing *RPS* computed with two alternative weightings, (*w*_anchor_:*w*_support_:*w*_synergy_) = 0.7:0.2:0.1 and 0.5:0.4:0.1.

**Figure S6.** ***In‑vitro* biotinylation activity of TurboID-CDNs.**

(A) Streptavidin-HRP blot for TurboID-positive/+biotin, TurboID-negative/+biotin, and TurboID-positive/no-biotin samples; ATP was supplied during all reactions. (B) SDS-PAGE of total protein indicating comparable loading. (C) Densitometric summary of lane-integrated streptavidin signals normalized to total protein. The comparison supports TurboID- and biotin-dependent labeling under ATP-supplemented conditions; ATP dependence was not independently tested.

**Figure S7. Venn diagrams summarizing identifications in heavy (recipient; red) and light (donor; blue) channels for mCherry‑positive cells.**

(A) TurboID‑CDNs, (B) TurboID‑sEVs.
